# Portable Fluorescence Microarray Reader-Enabled Biomarker Panel Detection System for Point-of-Care Diagnosis of Lupus Nephritis

**DOI:** 10.3390/mi16020156

**Published:** 2025-01-29

**Authors:** Aygun Teymur, Iftak Hussain, Chenling Tang, Ramesh Saxena, David Erickson, Tianfu Wu

**Affiliations:** 1 Department of Biomedical Engineering, University of Houston, Houston, TX 77024, USA; arteymur@cougarnet.uh.edu (A.T.); ctang9@central.uh.edu (C.T.); 2 Sibley School of Mechanical and Aerospace Engineering, Cornell University, Ithaca, NY 14850, USA; ih257@cornell.edu; 3 Department of Nephrology, University of Texas Southwestern Medical Center, Dallas, TX 75390, USA; ramesh.saxena@utsouthwestern.edu

**Keywords:** point-of-care (POC), portable microarray reader, lupus nephritis, diagnostics, biomarkers, disease monitoring

## Abstract

Point-of-care (POC) testing has revolutionized diagnostics by providing rapid, accessible solutions outside traditional laboratory settings. However, many POC systems lack the sensitivity or multiplexing capability required for complex diseases. This study introduces an LED-based fluorescence reader designed for POC applications, enabling multiplex detection of lupus nephritis (LN) biomarkers using a biomarker microarray (BMA) slide. The reader integrates an LED excitation source, neutral density (ND) filters for precise intensity control, and onboard image processing with Gaussian smoothing and centroid thresholding to enhance signal detection and localization. Five LN biomarkers (VSIG4, OPN, VCAM1, ALCAM, and TNFRSF1B) were assessed, and performance was validated against a Genepix laser-based scanner. The LED reader demonstrated strong correlation coefficients (r = 0.96–0.98) with the Genepix system for both standard curves and patient samples, achieving robust signal-to-noise ratios and reproducibility across all biomarkers. The multiplex format reduced sample volume and allowed simultaneous analysis of multiple biomarkers. These results highlight the reader’s potential to bridge the gap between laboratory-grade precision and POC accessibility. By combining portability, cost-effectiveness, and high analytical performance, this fluorescence reader provides a practical solution for POC diagnostics, particularly in resource-limited settings, improving the feasibility of routine monitoring and early intervention for diseases requiring comprehensive biomarker analysis.

## 1. Introduction

Lupus nephritis (LN) arises as a major complication of systemic lupus erythematosus (SLE), driven by immune-mediated damage to the kidneys that can lead to progressive renal failure [1]. SLE is an autoimmune disease characterized by multisystem inflammation and the production of autoantibodies, such as antinuclear antibodies (ANAa), which drive tissue damage [2]. LN, in particular, arises from immune complex deposition in renal tissues, leading to inflammation, glomerular damage, and eventual kidney failure if left untreated [3]. Early and accurate detection is vital to prevent irreversible renal damage, but traditional diagnostic approaches rely heavily on invasive renal biopsies or centralized laboratory assays, which limit accessibility and frequency of monitoring. To address these challenges, point-of-care (POC) testing offers a promising alternative by enabling rapid, portable, and cost-effective diagnostics.

POC testing has gained increasing attention in recent years, particularly following the COVID-19 pandemic, which underscored the need for accessible, rapid diagnostic tools [4]. While glucose monitoring has historically dominated the POC testing market, diagnostic and disease screening applications have become the fastest-growing sector due to demand for tools to monitor conditions such as cardiac diseases, cancers, and infectious diseases [5]. However, many POC devices are limited by their inability to measure multiple analytes simultaneously, reducing their efficiency and diagnostic utility in complex diseases. A multiplex POC device addresses these limitations by combining high-throughput biomarker detection with portability and cost-efficiency [6].

Fluorescence-based diagnostics are among the most sensitive and specific techniques for biomarker detection, widely used in molecular biology and clinical diagnostics [7,8]. Laboratory instruments, such as laser-based fluorescence scanners, offer exceptional precision and sensitivity but require specialized infrastructure, trained personnel, and significant financial investment [9]. These constraints have driven research efforts to develop portable, low-cost fluorescence systems capable of maintaining high performance while operating outside laboratory environments. Recent advances in light-emitting diode (LED) technology and open-source hardware have enabled the development of compact fluorescence readers that integrate optical excitation, detection, and data analysis into a single, user-friendly device [10].

One of the main challenges in fluorescence detection is the reliable quantification of signals, particularly in low-intensity contexts where noise and background interference can compromise accuracy. To address this challenge, our fluorescence reader incorporates an onboard image processing pipeline that automates key steps such as spot detection, segmentation, and intensity quantification. Gaussian smoothing can further be applied to reduce high-frequency noise by averaging pixel values with a Gaussian kernel [11]. In fluorescence imaging, Gaussian smoothing is particularly beneficial for improving signal-to-noise ratios, thereby facilitating more accurate detection of fluorescent signals. Centroid thresholding can also be employed to accurately localize the center of fluorescent spots within an image [12]. By integrating these processes through an onboard computational platform, our fluorescence reader eliminates the need for external computation and facilitates standalone operation.

To address the limitations of existing systems, we developed an LED-based fluorescence reader designed to detect LN biomarkers using a biomarker microarray (BMA) slide. The reader uses a high-power LED as the excitation source, which is collimated through an aspheric condenser lens to produce a uniform light beam optimized for Cy3 fluorophores. LEDs offer several advantages over lasers for POC applications, including lower cost, reduced power consumption, compact size, and compatibility with portable designs [13,14].

Based on our recent findings, five biomarkers (VSIG4, OPN, VCAM1, ALCAM, and TNFRSF1B) were selected for the development of the smartphone-based multiplexed biomarker detection platform for LN [15]. The slide format supports simultaneous detection of up to 16 patients for a panel of the selected markers. To validate the performance of the fluorescence reader, a series of experiments were conducted comparing its sensitivity, reproducibility, and diagnostic utility to a laser-based scanner, a widely recognized system for imaging microarrays.

This paper presents the design, calibration, and validation of the fluorescence reader, emphasizing its performance in detecting LN biomarkers. By demonstrating its sensitivity and reproducibility in comparison to established laser-based systems, we highlight the potential of this device to transform POC testing by bridging the gap between high-performance laboratory instruments and portable, cost-effective solutions.

## 2. Materials and Methods

### 2.1. Fluorescent Reader Development

**Figure 1:**
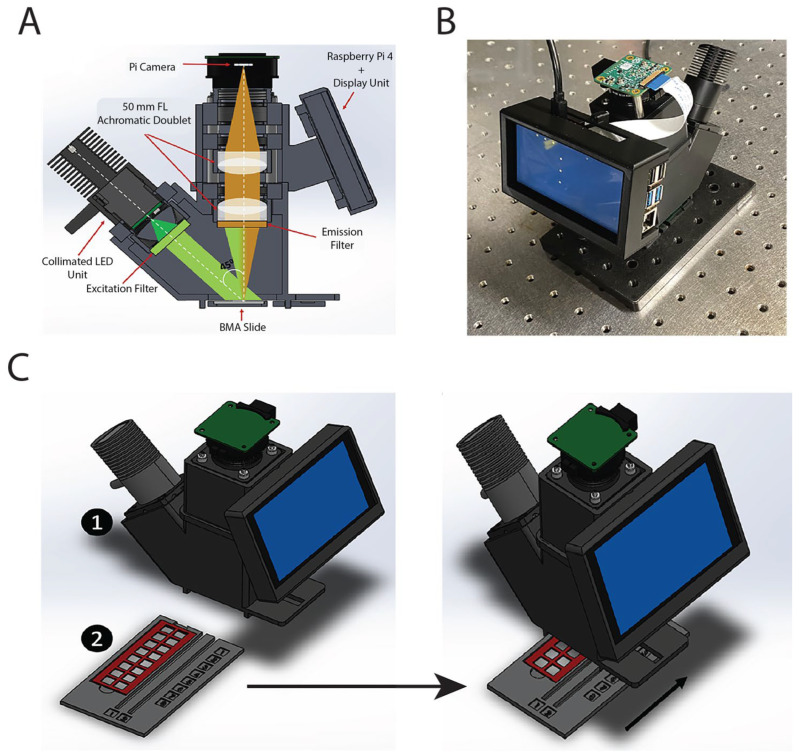
(**A**) Schematic of the fluorescence reader showing the LED excitation source, optical filters, BMA slide, and imaging components. (**B**) Photograph of the fully assembled fluorescence reader, including the integrated Raspberry Pi camera and display. (**C**) Illustration of the modular design, showing the BMA slide being inserted into the reader for fluorescence analysis. The number 1 represents the scanning device, while number 2 denotes the slide and its holder, which are positioned beneath the BMA reader for further analysis.

An affordable and portable fluorescence reader was developed for detecting LN biomarkers using a custom-developed BMA slide. The system integrates a compact optical assembly and computational platform to provide high-sensitivity fluorescence detection for a Cy3 fluorophore (Figure 1A). The optical design utilizes a high-power white light-emitting diode (LED) (400 nm–700 nm, 570 mW, MWWHL4; Thorlabs, Newton, MA, USA) as the excitation source, collimated through an aspheric condenser lens (ACL2520U, Thorlabs) to produce a uniform light beam. The LED is positioned at a 45° angle relative to the BMA slide surface, which helps minimize background noise and enhances the detection of low-intensity fluorescence signals. The excitation light is directed onto the BMA slide through total internal reflection (TIR) between the glass/air interfaces, ensuring efficient illumination of fluorescent spots.

To isolate the excitation wavelength for Cy3 fluorescent dye excitation, the collimated light beam passes through an excitation filter (535 nm center wavelength, 40 nm bandwidth, HPX535-40; Newport, Irvine, CA, USA). The emitted fluorescence signal from the BMA spots is filtered through an emission filter (610 nm center wavelength, 50 nm bandwidth, HPM610-50, Newport) to remove residual excitation light, and the resulting signal is captured by a high-resolution Raspberry Pi camera module (4056 × 3040 pixels, Sony IMX477 sensor; Sony Semiconductor, Atsugi, Japan). The optical system, comprising a pair of 50 mm focal length achromatic doublet lenses (AC254-050-A, Thorlabs), provides a field of view (FOV) of ~6.287 mm × 4.712 mm, sufficient to capture all the fluorescent spots within a single block of the BMA slide.

The BMA reader comprises two parts: the imaging module and the BMA slide holder (Figure 1C). The slide holder is designed for seamless integration into the reader through a slot at the base. When inserted, the BMA chamber aligns with the optical window of the fluorescence reader, facilitating consistent illumination and imaging (Figure 1C). The excitation power incident on the slide was determined to be sufficient at 250 mW for the excitation of fluorescent spots on the microarray. The system is controlled by a Raspberry Pi 4B board, which manages the camera module, captures fluorescence images, and performs on-board image processing and analysis through a custom Python-based application (Figure 1B). The camera parameters were optimized to maximize the signal-to-noise ratio (SNR) for the detection of low-intensity fluorescence signals. A 4.3-inch capacitive touch display with a DSI interface (800 × 480 pixels, WaveShare; Shenzhen, China) is integrated into the reader, providing a user-friendly interface for image display and analysis. The inclusion of this onboard display enables standalone operation without the need for an external computer.

### 2.2. Fabrication and Assembly of the BMA Chip

**Figure 2:**
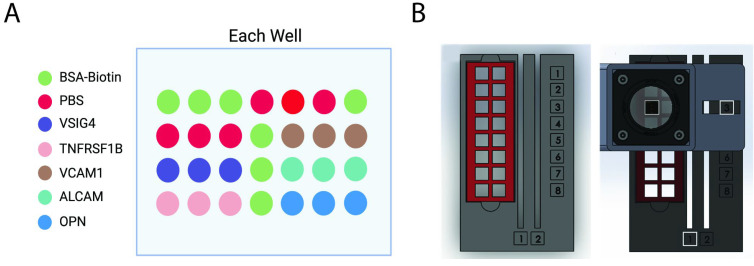
(**A**) Layout of the biomarker microarray (BMA) slide, showing the arrangement of capture antibodies and controls within each well. (**B**) Illustration of the BMA slide design with 16 wells and its alignment within the slide holder for analysis. The numbers along the side correspond to the row numbers of each well, while the numbers along the bottom represent the column positions of the slide.

Each well of the BMA slide consists of five printed capture antibodies, anti-VSIG4, anti-TNFRSF1B, anti-VCAM1, anti-ALCAM, and anti-OPN (Figure 2A). The slides were prepared using a non-contact microarray printing robot (sciFLEXARRAYER S3; Scienion GmbH, Berlin, Germany), which printed capture antibodies for each biomarker in triplicate at a controlled drop volume of 450 ± 20 pL. The printing process utilized a PDC90 needle (Scienion GmbH) and was conducted at 25 °C with a relative humidity of 60%. Following printing, the slides were left to dry overnight within the printing chamber and subsequently assembled with SecureSeal Hybridization Chambers (Grace Bio-Labs, Bend, OR, USA). Each slide consisted of 16 identical arrays, with the chambers designed to isolate individual arrays and prevent cross-contamination between samples. The assay protocol used for this array followed previously established methods [16]. After drying overnight, each chamber was then blocked with 40 µL of blocking buffer at room temperature for one hour. Following this step, the blocking buffer was removed, and pre-diluted recombinant proteins or serum samples were introduced into designated wells for a two-hour incubation. The slides were then thoroughly washed, incubated with streptavidin-HRP, and subjected to additional washes before being treated with Cy3-Streptavidin to detect fluorescent signals corresponding to the printed antibodies. After adding the streptavidin-Cy3, the fluorescent signals on the slide were read at an emission wavelength of 532 nm with a Genepix 4300A microarray scanner (Molecular Devices, San Jose, CA, USA) and quantified with Genepix Pro7.0 software (Molecular Devices, CA, USA). The BMA reader was further used to compare signal results with the Genepix scanner.

The slide consists of 16 wells, which align with the optical window of the BMA reader, ensuring the biomarker spots are within the imaging module’s FOV (Figure 2B). The housing for the imaging module and slide holder was fabricated using polylactic acid (PLA) filament (1.75 mm; Raise3D, Irvine, CA, USA) with a Raise3D Pro3 3D printer. All parts were designed in SolidWorks (Dassault Systèmes, Waltham, MA, USA). The reader’s final dimensions were 5.5 × 3.5 × 2 inches, with a total weight of 250 g.

### 2.3. Image Analysis and Processing Pipeline

**Figure 3:**
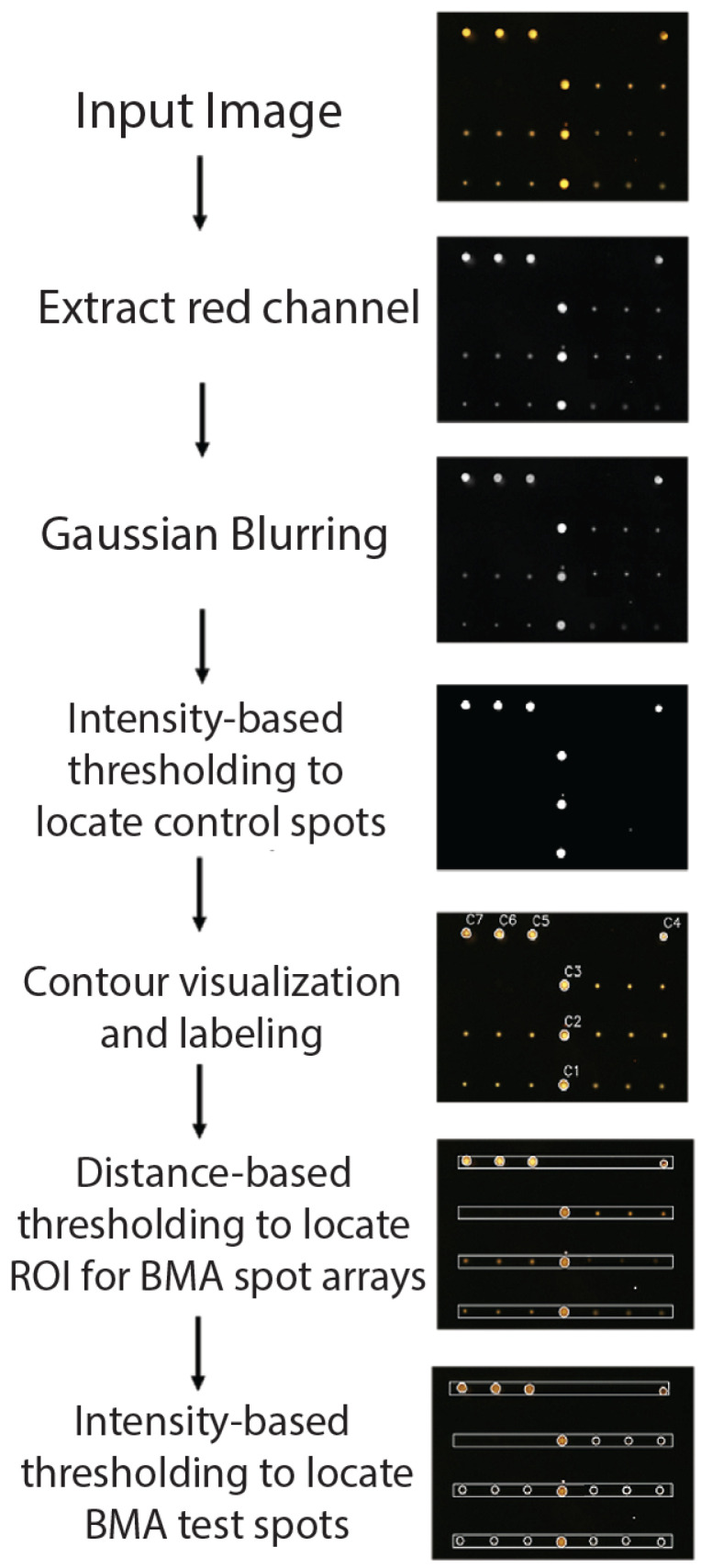
Workflow for image processing, including red channel extraction, Gaussian blurring, intensity-based thresholding for control spot detection, contour labeling, and localization of test spots using distance-based thresholding.

An image processing pipeline was developed to automate camera parameter control, spot detection, and intensity analysis for the BMA slide using Python (Figure 3). Fluorescence images were imported in ‘png’ format, and the red channel of the RGB images was used for analysis due to its dominant contribution to fluorescence intensity.

Gaussian blurring with a 9 × 9 kernel was applied to reduce noise and provide a smoother image. Intensity-based thresholding was then used to identify control spots. To exclude non-specific spots, circular contours with areas smaller than 100 pixels were filtered out, based on the experimentally determined minimum diameter of control spots. The remaining contours were labeled, and their centroids were calculated to establish precise control spot locations. The predefined coordinates from the automated dispensing system ensured consistent placement of spots on the slide. Using the centroids of the control spots (C1, C2, and C3), test spot locations were determined within a 1200-pixel range on either side, bounded by control spots C4 and C7. Regions of interest (ROIs) corresponding to the test spots were identified, and the same image processing steps were applied to detect their locations. Average fluorescence intensity values were calculated for each identified spot.

### 2.4. Patients, Clinical Samples, and Reagents

Serum samples from 10 healthy individuals and 10 LN patients were selected for diagnostic protein microarray analysis. The LN patients were classified as having a Systemic Lupus Erythematosus Disease Activity Index (SLEDAI) score greater than 4 and a renal SLEDAI (rSLEDAI) score above 0. The rSLEDAI focuses on renal-related manifestations of SLE, including hematuria (more than 5 red blood cells per high-power field), pyuria (over 5 white blood cells per high-power field), proteinuria (exceeding 0.5 g per 24 h), and the presence of urinary casts. All procedures involving human subjects were conducted in accordance with the institutionally approved IRB protocol (University of Houston, IRB #STUDY00001299). The clinical samples utilized in this study were sourced from the existing sample bank at the University of Houston.

Five protein biomarkers were used in the array in this study. Osteopontin (OPN, catalog no. DY1433), activated leukocyte cell adhesion molecule (ALCAM, catalog no. DY656), vascular cell adhesion molecule 1 (VCAM1, catalog no. DY809), V-set and immunoglobulin domain containing-4 (VSIG4, catalog no. MAB46461-100), and TNF receptor superfamily member 1B (TNFRSF1b, catalog no. DY726) proteins were utilized as capture antibodies and standards and were purchased from R&D Systems (Minneapolis, MN, USA). Additionally, 100 μg/mL BSA-Biotin (Thermo Scientific, Waltham, MA, USA) and 1X PBS buffer (R&D Systems) were used as a positive control and a negative control, respectively. The epoxy-modified polymer slides (STRATEC Consumables GmbH, Birkenfeld, Germany) were used for the immobilization of the antibodies and their subsequent detection.

For the detection of these proteins, a cocktail mixture of biotinylated antibodies was used for all targets except VSIG4, for which a separately purchased antibody (catalog no. BAF4646) was employed. Biotinylated anti-human IgG (catalog no. 109-065-170) was also utilized in the mixture and purchased from Jackson ImmunoResearch (West Grove, PA, USA). Additionally, 10 µL of the serum samples were diluted with Super G blocking buffer (catalog no. 105101) from Grace Bio-Labs (Bend, OR, USA) at an optimized dilution ratio. Streptavidin-Cy3 dye was used (Jackson ImmunoResearch) for fluorescent detection. The assay required a total runtime of 5 h. Furthermore, 4-parameter logistic (4PL) standard curves were generated based on serially diluted protein standards to determine the protein concentrations in the patient serum.

### 2.5. Statistical Analysis

Images generated by the BMA reader were processed and analyzed using a custom Python script. All patient data were analyzed, plotted, or visualized with the R 4.1.0 language or ggplot2 package [17]. Groupwise comparisons of statistical significance (assessed with *p* values) were performed using a *t*-test, unless stated otherwise. AUC analysis was performed using the pROC package [18]. The correlation between biomarker levels and clinical or pathologic parameters was determined using Spearman’s correlation coefficients and the COR package [19].

## 3. Results

### 3.1. Power Assessment to Excite BMA Spots

**Figure 4:**
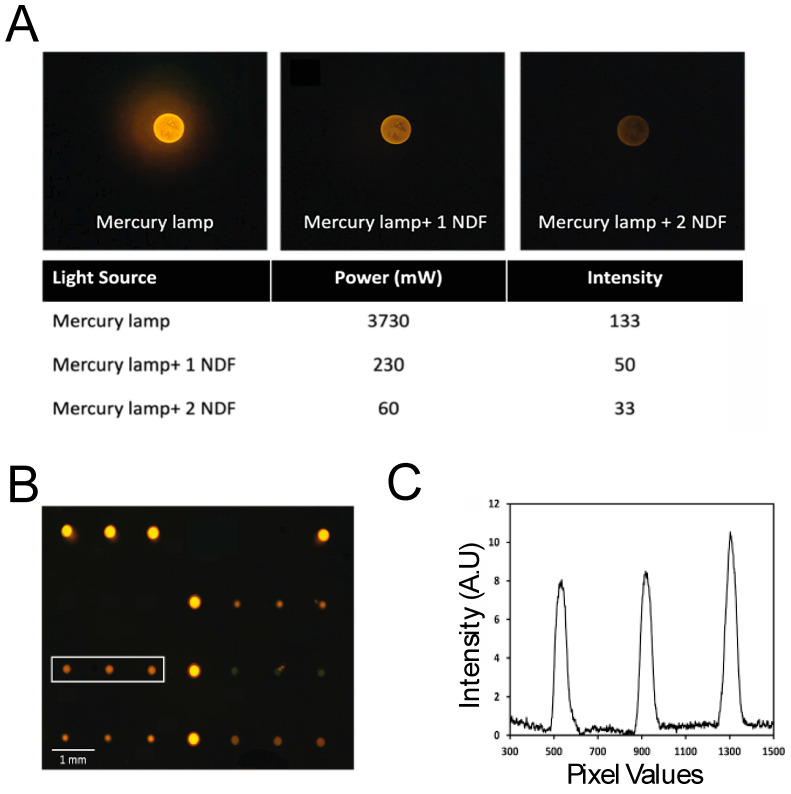
(**A**) Comparison of fluorescence spot intensity under a mercury lamp with and without neutral density filters (NDFs), alongside the corresponding power and intensity measurements. (**B**) Fluorescence image showing distinct spots on the BMA slide, with a highlighted region for further analysis. (**C**) Intensity profile across the highlighted region, showing fluorescence peaks corresponding to individual spots.

The power measurement was performed using an inverted fluorescence microscope (Olympus IX51) equipped with a high-power mercury lamp and a fluorescence filter set specific for the Cy3 fluorophore. A control spot was imaged at 40× magnification under full system power to establish baseline signal intensity (Figure 4A).

The incident power was subsequently reduced using neutral density filters (NDFs) (U-25ND25, Olympus), and the images were captured. It was found that with two NDFs, the spot is barely visible. We measured the intensity values of the same spot captured in three different configurations and found that a minimum power of 230 mW is needed to clearly visualize the mini-array spots. The light source was selected based on this information when developing the fluorescent reader. Fluorescence images confirmed that all spots on the mini-array slide were distinctly visible, and intensity profiles were generated to evaluate fluorescence signal distribution (Figure 4B). Pixel intensity peaks corresponded to individual spots (Figure 4C), showing that despite low absolute intensity values, the system achieved a high signal-to-noise ratio (SNR) sufficient to reliably distinguish spots from the background.

**Figure 5:**
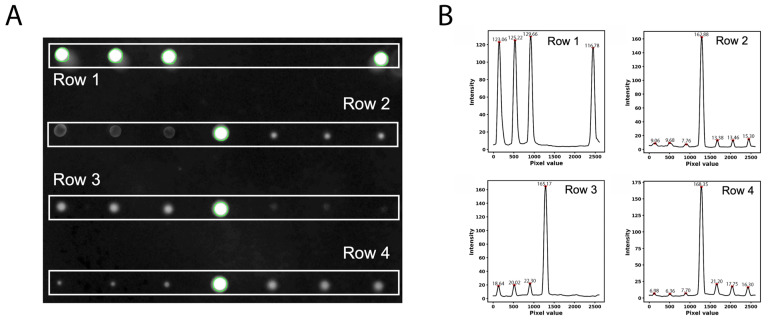
(**A**) Fluorescence image of the BMA slide showing individual rows (Row 1–4) with segmented fluorescence spots. (**B**) Intensity profiles for each row, with peaks corresponding to fluorescence signals from the detected spots.

The peaks are generated by segmenting the fluorescence image into four distinct rows, with each row representing a set of printed spots (Figure 5A). Intensity profiles are created by analyzing pixel values across a defined area within each row. This segmentation ensures that fluorescence signals are isolated and evaluated independently for each row to minimize the overlap or interference between adjacent spots. Peaks in the intensity profile represent individual fluorescence signals, with the height of each peak corresponding to the fluorescence intensity of the respective spot (Figure 5B). Background pixel values are subtracted to enhance the accuracy of the measurements, ensuring that the profiles reflect only the true signal from the fluorescently labeled spots.

### 3.2. Performance Comparison Between BMA Reader and Genepix Scanner

**Figure 6:**
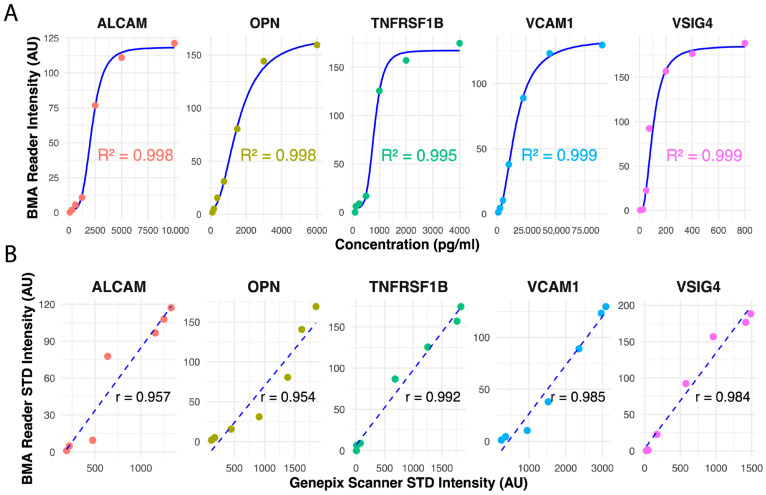
(**A**) Standard curves for the BMA reader showing fluorescence intensity (AU) versus biomarker concentration, with R^2^ values indicating strong correlation for all biomarkers. (**B**) Comparison of standard intensities between the BMA reader and Genepix scanner, with corresponding Pearson correlation coefficients (r) for all biomarkers.

Standard curves were generated for each of the five biomarkers (ALCAM, OPN, TNFRSF1B, VCAM1, and VSIG4) using a four-parameter logistic (4PL) regression model to fit the fluorescence intensity data obtained from the BMA reader. The performance of the BMA reader was evaluated against the Genepix scanner, a widely recognized standard scanner for fluorescence microarray imaging. For the BMA reader, the curves demonstrated strong relationships between the standard protein concentrations and fluorescence intensity for ALCAM, OPN, TNFRSF1B, VCAM1, and VSIG4, with coefficients of determination (R^2^) of 0.998, 0.998, 0.995, 0.999, and 0.999, respectively (Figure 6A). Similarly, the Genepix scanner exhibited high R^2^ values of 0.986, 0.998, 0.996, 0.997, and 0.996 for the same biomarkers (Supplementary Figure S2A). The fluorescence intensity values measured by the BMA reader were directly compared to those obtained using the Genepix scanner, which serves as the reference standard for fluorescent array detection. Pearson correlation coefficients for the standard samples demonstrated strong correlation between the two platforms, with r-values of 0.957, 0.954, 0.992, 0.985, and 0.984 for ALCAM, OPN, TNFRSF1B, VCAM1, and VSIG4, respectively (Figure 6B). Representative scans for the varying concentrations of standard curves are provided in Supplementary Figure S1. Similarly, patient sample fluorescence intensities showed a strong correlation between the BMA reader and the Genepix scanner, with r-values of 0.97, 0.96, 0.93, 0.94, and 0.92 for the same biomarkers (Supplementary Figure S2C).

### 3.3. BMA Multiplex Biomarker Analysis in Patient Samples

**Figure 7:**
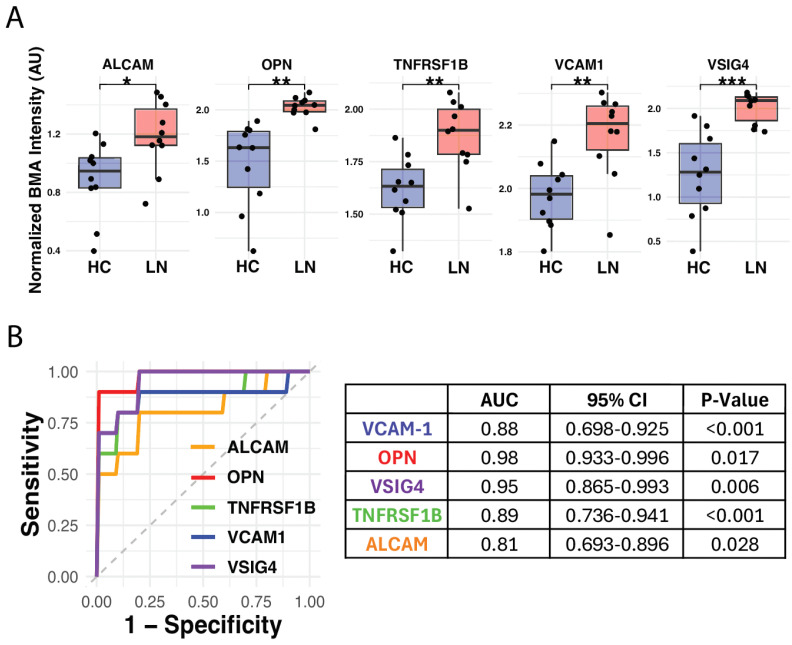
(**A**) Boxplots comparing normalized BMA intensity for biomarkers between healthy controls (HCs) and lupus nephritis (LN) patients, with statistical significance indicated as: * *p* < 0.01; ** *p* < 0.001; *** *p* < 0.0001. (**B**) Receiver operating characteristic (ROC) curves and area under the curve (AUC) values for the 5 biomarkers and an overall score, highlighting their diagnostic performance in distinguishing HCs from LN patients.

Normalized fluorescence intensity values for the five biomarkers (ALCAM, OPN, TNFRSF1B, VCAM1, and VSIG4) were compared between healthy controls (HCs) and lupus nephritis (LN) patient groups. Significant differences in fluorescence intensity were observed for all biomarkers, with LN patients exhibiting elevated levels compared to healthy controls (Figure 7A). Boxplots from the Genepix scanner (Supplementary Figure S2B) similarly demonstrated significantly elevated biomarker levels in LN patients compared to HCs. Notably, OPN and VSIG4 displayed the largest differences in normalized intensity, highlighting their potential as robust biomarkers for LN detection.

Receiver operating characteristic (ROC) curve analysis was performed to evaluate the diagnostic performance of each biomarker in distinguishing LN patients from healthy controls. Area under the curve (AUC) values were calculated, demonstrating high diagnostic accuracy for all biomarkers (Figure 7B). OPN and VSIG4 achieved the highest AUC values of 0.98 (95% CI: 0.933–0.996, *p* = 0.017) and 0.95 (95% CI: 0.865–0.993, *p* = 0.006), respectively. TNFRSF1B and VCAM1 also exhibited strong diagnostic performance with AUC values of 0.89 (95% CI: 0.736–0.941, *p* < 0.001) and 0.88 (95% CI: 0.698–0.925, *p* < 0.001), respectively. ALCAM showed an AUC of 0.81 (95% CI: 0.693–0.896, *p* = 0.028), demonstrating moderate diagnostic utility. Overall, the combined biomarker panel yielded an AUC of 0.92 (95% CI: 0.883–0.971, *p* = 0.004), highlighting the potential of this multiplexed approach for LN diagnosis. AUC values for the Genepix scanner were 0.99 for VCAM1, 0.97 for OPN, 0.99 for VSIG4, 0.85 for TNFRSF1B, and 0.91 for ALCAM, with an overall AUC of 0.94 (Supplementary Figure S2D). These results align closely with the AUC values obtained from the BMA reader.

## 4. Discussion

Systemic lupus erythematosus (SLE) is an autoimmune disease marked by widespread microvascular inflammation affecting multiple organ systems and the production of various autoantibodies, most notably antinuclear antibodies (ANAs) [20,21]. Lupus nephritis (LN) is a severe manifestation of SLE, characterized by immune complex deposition in the kidneys, leading to inflammation and potential renal failure [22]. While renal disease often manifests within the first year of SLE diagnosis and nearly always within five years, it can also appear as the initial symptom or emerge at any stage throughout the disease’s course [23]. Early and accurate detection is crucial to prevent irreversible damage. Traditional diagnostic methods, such as renal biopsy, are invasive and carry inherent risks, underscoring the need for non-invasive, reliable, and efficient diagnostic tools [24].

In recent years, the development of portable fluorescence readers has garnered significant attention in biomedical diagnostics. These devices offer a cost-effective and accessible alternative to standard laboratory-based equipment, enabling point-of-care testing and facilitating timely clinical decision-making. The integration of advanced optical components, such as collimated LED light sources and high-resolution imaging systems, has enhanced the sensitivity and specificity of these readers in detecting various biomarkers [25]. For instance, a study demonstrated the use of a portable microplate reader that supports colorimetric, fluorescence, and luminescence analyses for the detection of SARS-CoV-2 N-protein in a standard assay [26]. Of all optical detection techniques, fluorescence detection is the most extensively utilized in biological research and applications. Fluorescent detection offers superior sensitivity compared to colorimetric methods, allowing for the detection of lower analyte concentrations and improved dynamic range in assays [27]. Additionally, fluorescence provides greater signal-to-noise ratios and the ability to multiplex by using different fluorophores, making it more versatile for complex biological analyses [28,29]. A fluorescent multiplexed bead-based assay using flow cytometry demonstrated strong correlation with traditional single-analyte ELISA methods for detecting autoantibodies to SSA, SSB, Sm, and RNP, biomarkers frequently elevated in patients with SLE [30]. Another research group analyzed the components and clinical biomarkers found in urine, saliva, and sweat using aggregation-induced emission fluorogens [31].

The image processing pipeline designed for this fluorescence reader ensures consistent and reproducible quantification of fluorescence intensities. By employing Gaussian smoothing and centroid-based thresholding, the pipeline minimizes noise and maximizes detection accuracy for low-intensity spots. This method enhances the reliability of fluorescence measurements by isolating true signal intensities from background artifacts, a common challenge in multiplex biomarker quantification. Furthermore, the combination of smoothing and precise thresholding allows for the accurate differentiation of closely spaced fluorescence spots. Studies have demonstrated that Gaussian smoothing significantly enhances the reproducibility of fluorescence intensity measurements [32,33,34]. Meanwhile centroid-based thresholding improves spot detection accuracy by focusing on geometric center of signal contours in the presence of high noise or uneven spot reflections, especially in multiplex assays that require multiple spots [35]. This makes it highly effective in minimizing errors caused by uneven intensity distribution or small variations in spot alignment.

This method addresses limitations observed in other approaches that are based on identifying the lowest intensity values within the grayscale image but often fail to account for background variations or interferences inherent to clinical samples [36]. In another approach, Otsu’s method is an adaptive global thresholding approach that determines a threshold value to maximize the variance between foreground and background pixel intensities using bimodal thresholding [37]. While effective for general segmentation tasks, Otsu’s method may struggle with non-bimodal fluorescence images where signal intensities vary widely across spots, leading to the potential misclassification of weak signals as background or inclusion of noise as signal. Studies have shown that noise reduction through Gaussian blurring can improve the reproducibility of fluorescence signals in point-of-care diagnostics [38,39].

The thresholding employed in this system further enhances the BMA’s specificity. The algorithm considers the geometric center of the fluorescence signal contour and aligns it with predefined spatial coordinates for each biomarker. Additionally, the combination of area-based filtering ensures that noise artifacts, such as dust particles or nonspecific binding, are excluded. This is particularly important for multiplex systems, where minor misalignments can compromise biomarker quantification. The row-by-row approach enables systematic identification and quantification of fluorescence intensity across the array. Variations in peak intensity are seen within each row and are indicative of differences in fluorescence signal strength because of differences in biomarker concentration or spot quality. The reproducibility of peak profiles across multiple rows and slides further validates the performance of the reader and the uniformity of the printed mini-array spots.

Furthermore, the imaging system utilizes an LED-based system to excite the fluorophores on the surface of the BMA slide. We compared our BMA system to the Genepix platform that is a laser scanner using highly focused monochromatic light sources to achieve excitation. However, the inherent complexity and high cost of laser systems, along with their bulkiness and need for precise alignment, limit their practicality in point-of-care settings. In contrast, LEDs are compact, energy-efficient, and generate less heat, simplifying device design and maintenance [40,41]. LED-based systems offer a broad-spectrum excitation source that can be tailored with filters to match specific fluorophores, providing flexibility and cost-efficiency. We implemented neutral density filters to control the intensity of the LED light source without altering the spectral output. This broad-spectrum attenuation allows for precise control of the light intensity without altering the excitation spectrum required for a Cy3 fluorophore. We demonstrated that the device exhibits a strong correlation between laser-based and LED-based systems, indicating that its performance is comparable to established laser-based platforms.

This study demonstrates a fluorescence reader’s capability to provide measurements comparable to those of widely used platforms such as the Genepix scanner, with correlation coefficients exceeding 0.95. These strong correlations demonstrate that the BMA reader provides fluorescence intensity measurements comparable to the Genepix scanner, validating its performance as a reliable alternative for fluorescence-based biomarker analysis. The significance of this lies not only in diagnostic accuracy but also in portability and accessibility. Unlike the Genepix scanner, which requires specialized infrastructure and trained laboratory personnel, this reader integrates image acquisition and analysis into a single, user-friendly platform by utilizing a Raspberry Pi board as a digital screen. This distinction makes the device more suitable for decentralized healthcare settings. Existing portable fluorescence readers, while promising, often lack advanced image processing features. For instance, a study highlighted the limitations of fluorescence immunochromatographic assay readers that rely on basic image processing methods, emphasizing that the limited gray level of standard image sensors can restrict both the detection range and accuracy [42]. Similarly, the implementation of centroid thresholding in the BMA system prioritizes signal accuracy by isolating and analyzing the true fluorescence signal contours rather than simply summing raw intensity values.

The inclusion of a multiplex biomarker system for LN diagnostics enhances the device’s relevance. The five biomarkers chosen—ALCAM, OPN, TNFRSF1B, VCAM1, and VSIG4—are integral to the inflammatory and immune pathways implicated in LN. The ability to simultaneously detect these markers on a single platform allows clinicians to assess disease activity and severity comprehensively. While traditional single-analyte assays like ELISA remain standard in many settings, they lack the efficiency and throughput provided by multiplex assays. The simultaneous quantification of these biomarkers allows for the exploration of synergistic effects and co-expression patterns. For example, the concurrent elevation of adhesion molecules (ALCAM and VCAM1) alongside pro-inflammatory cytokines (TNFRSF1B and OPN) could provide deeper insights into endothelial dysfunction and immune activation in LN [43]. Such multiplexed insights are critical in stratifying patient treatments, especially in diseases with heterogeneity like SLE.

The selected biomarkers—ALCAM, OPN, TNFRSF1B, VCAM1, and VSIG4—have previously been found to be strongly associated with the pathophysiology of LN. ALCAM and VCAM1 mediate leukocyte adhesion and transmigration across the endothelium, processes that are heightened in LN due to persistent inflammation and endothelial activation [44]. ALCAM and VCAM1 were found to be significantly higher in LN patients at the time of nephritic flare and lower at the time of remission while also correlating with clinico-pathological parameters [45]. Elevated levels of these adhesion molecules in SLE patients correlate with disease activity and kidney injury while impairing endothelial function [46]. OPN, a multifunctional cytokine, has been extensively documented in LN, with elevated OPN levels serving as a marker of active renal disease [47,48,49]. Additionally, increased polymorphisms in TNFRSF1B alleles were significantly associated with an increased risk of SLE [50]. We have previously identified VSIG4 as a potential biomarker for LN diagnosis due to its significant upregulation in serum and its detection in renal tissue [51].

Multiplex platforms offer significant advantages over single-analyte assays by capturing the interplay of multiple biomarkers in a single assay. This multiplex array approach reduces assay time and sample volume when compared to traditional methods, such as ELISA, while maintaining comparable performance (Figure 6 and Figure 7 and Supplementary Figure S2). Multiplex detection has been applied in various diagnostic contexts, but its adoption in LN diagnostics remains limited. The reader’s ability to generate standard curves with R^2^ values exceeding 0.99 for all biomarkers highlights its precision and reproducibility. These results confirm the reader’s ability to accurately quantify fluorescence signals over a broad range of concentrations, making it suitable for biomarker detection in clinical samples. Furthermore, its ROC analysis demonstrated AUC values above 0.85 for all biomarkers, with combined performance reaching 0.92, underscoring its potential as a diagnostic tool.

In addition to its technical performance, the system offers significant advantages in terms of cost and practicality compared to similar commercial platforms. For instance, the GenePix scanner, a widely used laser-based fluorescence reader, is highly sensitive but comes with substantial costs, complex infrastructure requirements, and the need for trained personnel, limiting its feasibility for point-of-care applications. In contrast, the LED-based system described in this study is compact, cost-efficient, and user-friendly, making it well-suited for decentralized healthcare settings. However, it is important to acknowledge that while the LED-based system provides comparable performance for fluorescence intensity measurements, it may have limitations in throughput and resolution compared to more advanced laser-based systems.

The combination of advanced image processing, high biomarker specificity, and multiplex capability positions this reader as a transformative tool in LN diagnostics. Beyond LN, this platform could be adapted for other autoimmune or inflammatory conditions by incorporating disease-specific biomarkers. Future work could focus on increasing the dynamic range of detection and integrating additional computational features, such as machine learning algorithms, for automated data interpretation. Validation studies were conducted using a small patient cohort and further clinical validation with larger patient populations is needed.

## 5. Conclusions

In conclusion, the development of a portable fluorescence reader capable of multiplex biomarker detection represents a significant advancement in the non-invasive diagnosis and monitoring of lupus nephritis. Its high correlation with standard laboratory equipment, combined with its portability and cost-effectiveness, positions it as a valuable tool in both clinical and research settings. By facilitating early detection and personalized treatment strategies, this device has the potential to improve patient outcomes and advance the management of various diseases.

## Data Availability

The original contributions presented in this study are included in the article/Supplementary Materials. Further inquiries can be directed to the corresponding authors.

## Supplementary Materials

The following supporting information can be downloaded at: https://www.mdpi.com/article/10.3390/mi16020156/s1, Figure S1: The left panel represents a scan performed using the GenePix Pro scanner, while the right panel showcases a scan obtained with the BMA reader. Each column shows the standard curves for each biomarker, with concentrations ranging from high (top rows) to low (bottom rows). The concentrations of each biomarker are indicated on the standard curves for the GenePix Pro scanner and the BMA reader.; Figure S2: (A) Standard curves generated using the Genepix scanner for all five biomarkers (ALCAM, OPN, TNFRSF1B, VCAM1, and VSIG4), showing corresponding R² values. (B) Boxplots comparing normalized Genepix intensities for healthy controls (HC) and lupus nephritis (LN) patients across all biomarkers. Significant differences in biomarker levels are indicated, with LN patients consistently showing elevated levels (*p* < 0.05). (C) Correlation analysis between the Genepix scanner and the BMA reader for patient samples. Pearson correlation coefficients (r) are displayed for each biomarker. (D) Receiver operating characteristic (ROC) curves for each biomarker, highlighting their diagnostic per-formance in distinguishing HC from LN patients. Area under the curve (AUC) values are presented in the accompa-nying table, showing diagnostic accuracy for all biomarkers.
