# Supplementary material for: Portable Fluorescence Microarray Reader-Enabled Biomarker Panel Detection System for Point-of-Care Diagnosis of Lupus Nephritis
Source: Micromachines (Basel). 2025 Jan 29;16(2):156. doi: 10.3390/mi16020156 (PMC11857597; doi:10.3390/mi16020156)
Supplement: Supplementary file 1 [file micromachines-16-00156-s001.zip › micromachines-3386312-supplementary.pdf]

micromachines

Supplementary

Portable Fluorescence Microarray Reader-Enabled Biomarker Panel Detection System for Point-Of-Care Diagnosis of Lupus Nephritis

Aygun Teymur [1,†], Iftak Hussain [2,†], Chenling Tang [1], Ramesh Saxena [3],David Erickson [2,*] and Tianfu Wu [1,*]

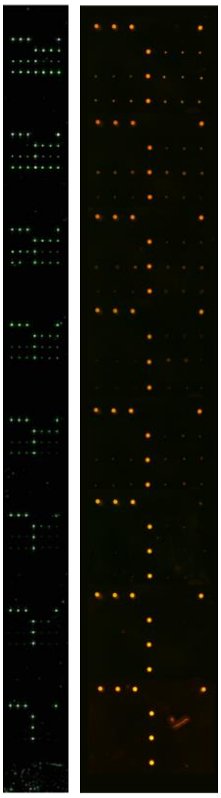

Supplementary Figure S1. The left panel represents a scan performed using the GenePix Pro scanner, while the right panel showcases a scan obtained with the BMA reader. Each column shows the standard curves for each biomarker, with concentrations ranging from high (top rows) to low (bottom rows). The concentrations of each biomarker are indicated on the standard curves for the GenePix Pro scanner and the BMA reader.

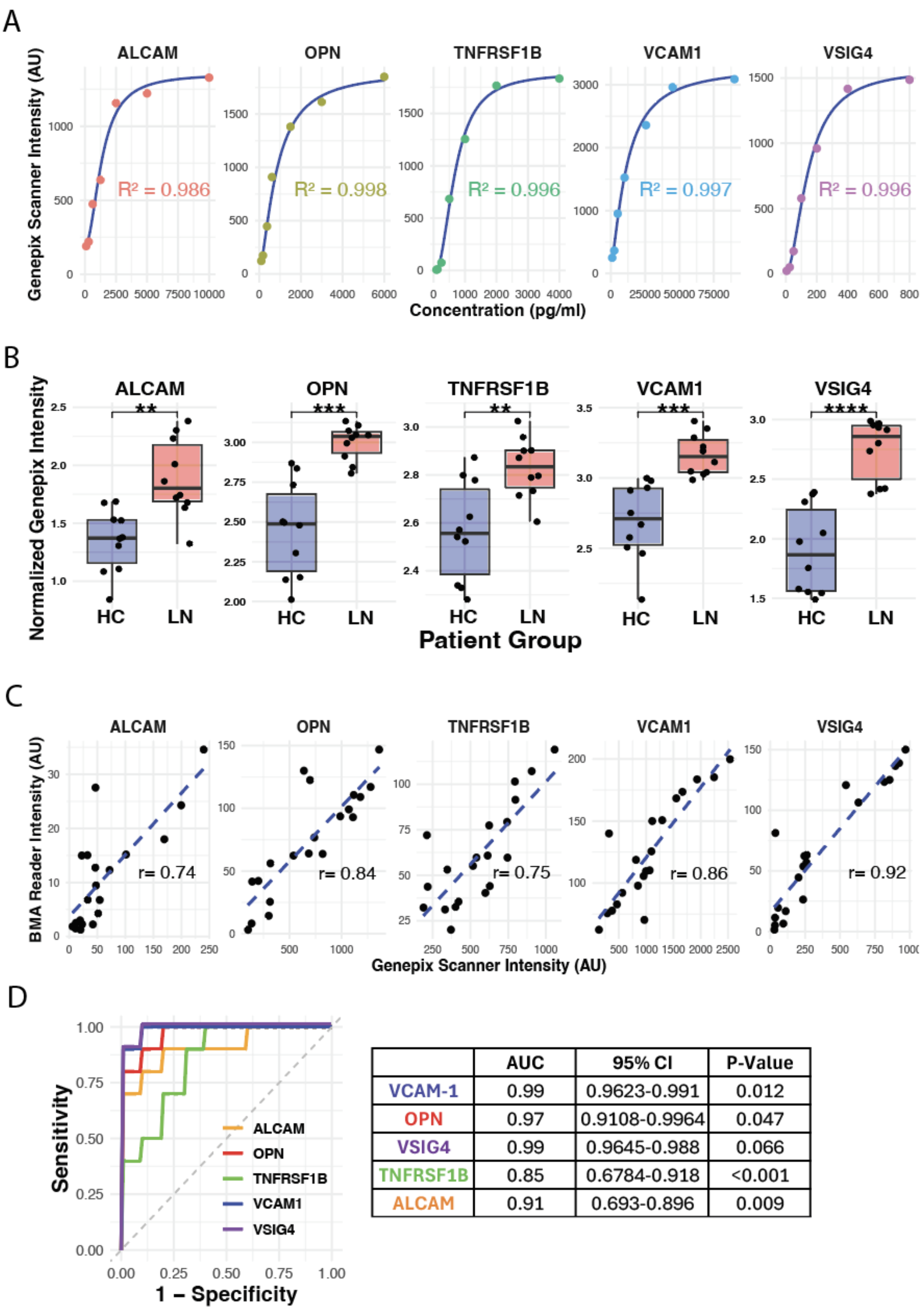

	AUC	95% CI	P-Value
VCAM-1	0.99	0.9623-0.991	0.012
OPN	0.97	0.9108-0.9964	0.047
VSIG4	0.99	0.9645-0.988	0.066
TNFRSF1B	0.85	0.6784-0.918	<0.001
ALCAM	0.91	0.693-0.896	0.009

Supplementary Figure S2. (A) Standard curves generated using the Genepix scanner for all five biomarkers (ALCAM, OPN, TNFRSF1B, VCAM1, and VSIG4), showing corresponding R^2 values. (B) Boxplots comparing normalized Genepix intensities for healthy controls (HC) and lupus nephritis (LN) patients across all biomarkers. Significant differences in biomarker levels are indicated, with LN patients consistently showing elevated levels ($p < 0.05$). (C) Correlation analysis between the Genepix scanner and the BMA reader for patient samples. Pearson correlation coefficients (r) are displayed for each biomarker. (D) Receiver operating characteristic (ROC) curves for each biomarker, highlighting their diagnostic performance in distinguishing HC from LN patients. Area under the curve (AUC) values are presented in the accompanying table, showing diagnostic accuracy for all biomarkers.
